# Do not judge a book by its cover: would *Triatoma tibiamaculata* (Pinto, 1926) belong to *Triatoma* Laporte, 1832, or to *Panstrongylus* Berg, 1879, with misleading homoplasies?

**DOI:** 10.1186/s13071-022-05314-7

**Published:** 2022-05-28

**Authors:** Isadora Freitas Bittinelli, Jader de Oliveira, Yago Visinho dos Reis, Amanda Ravazi, Fernanda Fernandez Madeira, Ana Beatriz Bortolozo de Oliveira, Giulia Montanari, Ana Julia Chaves Gomes, Laura Poloto Cesaretto, Isabella da Silva Massarin, Cleber Galvão, Maria Tercília Vilela de Azeredo-Oliveira, João Aristeu da Rosa, Kaio Cesar Chaboli Alevi

**Affiliations:** 1 Universidade Estadual Paulista “Júlio de Mesquita Filho” (UNESP), Instituto de Biociências Rua Dr. Antônio Celso Wagner Zanin, 250, Distrito de Rubião Júnior, 18618-689 Botucatu, SP Brasil; 2 Laboratório de Entomologia em Saúde Pública, Departamento de Epidemiologia, Faculdade de Saúde Pública, Universidade de São Paulo (USP), Av. Dr. Arnaldo 715, São Paulo, SP Brasil; 3 Laboratório de Parasitologia, Universidade Estadual Paulista “Júlio de Mesquita Filho” (UNESP), Faculdade de Ciências Farmacêuticas, Rodovia Araraquara-Jaú km 1, 14801-902 Araraquara, SP Brasil; 4 Laboratório de Biologia Celular, Universidade Estadual Paulista “Júlio de Mesquita Filho” (UNESP), Instituto de Biociências, Letras e Ciências Exatas, Rua Cristóvão Colombo 2265, 15054-000 São José do Rio Preto, SP Brasil; 5 Laboratório Nacional e Internacional de Referência em Taxonomia de Triatomíneos, Instituto Oswaldo Cruz (FIOCRUZ), Av. Brasil 4365, Pavilhão Rocha Lima, sala 505, 21040-360 Rio de Janeiro, RJ Brasil

**Keywords:** Chagas disease vector, Triatomines, Taxonomy, *Panstrongylus tibiamaculatus* comb. nov

## Abstract

**Background:**

*Triatoma tibiamaculata* is a species distributed in ten Brazilian states which has epidemiological importance as it has already been found infecting household areas. The taxonomy of this triatomine has been quite unstable: it was initially described as *Eutriatoma tibiamaculata*. Later, the species was transferred from the genus *Eutriatoma* to *Triatoma*. Although included in the genus *Triatoma*, the phylogenetic position of *T. tibiamaculata* in relation to other species of this genus has always been uncertain once this triatomine was grouped in all phylogenies with the genus *Panstrongylus*, rescuing *T. tibiamaculata* and *P. megistus* as sister species. Thus, we evaluated the generic status of *T. tibiamaculata* using phylogenetic and chromosomal analysis.

**Methods:**

Chromosomal (karyotype) and phylogenetic (with mitochondrial and nuclear markers) analyses were performed to assess the relationship between *T. tibiamaculata* and *Panstrongylus* spp.

**Results:**

The chromosomal and phylogenetic relationship of *T. tibiamaculata* and *Panstrongylus* spp. confirms the transfer of the species to *Panstrongylus* with the new combination: *Panstrongylus tibiamaculatus*.

**Conclusions:**

Based on chromosomal and phylogenetic characteristics, we state that *P. tibiamaculatus* comb. nov. belongs to the genus *Panstrongylus* and that the morphological features shared with *Triatoma* spp. represent homoplasies.

**Graphical Abstract:**

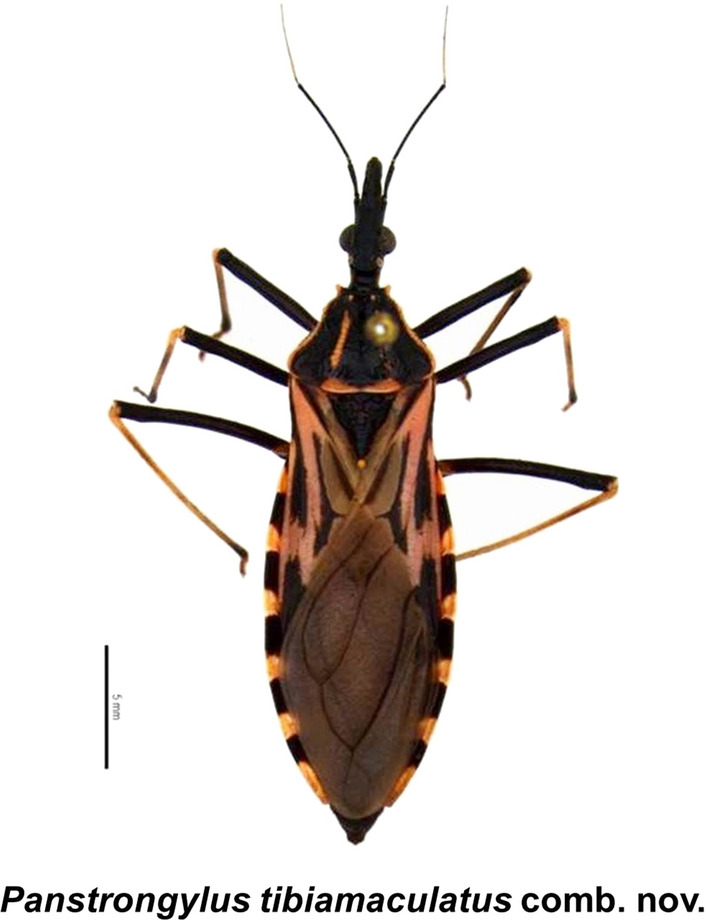

## Background

The members of the subfamily Triatominae (Hemiptera, Reduviidae) are hematophagous insects of great epidemiological importance as they act as vectors of the protozoan *Trypanosoma cruzi* (Chagas, 1909) (Kinetoplastida, Trypanosomatidae), the etiological agent of Chagas disease [1]. Chagas disease is a neglected disease that affects about 8 million people and puts another approximately 25 million at risk of infection [1]. The main way to minimize the incidence of new cases is based on the control of vector populations [1], the studies related to these insects being of extreme importance for public health once they can generate results to help vector control programs in the prophylaxis of Chagas disease.

Systematics has contributed to the correct identification of triatomines and consequently to the surveillance activities of vector control programs [2, 3]. However, in the face of evolutionary events (cryptic speciation and phenotypic plasticity [4]) and associated taxonomic problems, in most cases, with classical taxonomy [5, 6] (based on the morphological characterization of the species [3, 6]), > 190 synonymizations have occurred in the Triatominae subfamily [7]. This highlights the importance of integrative taxonomy for the description of new species [6], as performed by Dorn et al. [8], Lima-Cordón et al. [9] and Alevi et al. [10].

Currently, 157 species are described in the subfamily Triatominae (with 154 extant species and three fossil species), grouped into 18 genera and 5 tribes [6–12]. In Brazil, > 60 species are distributed among the following genera: *Alberprosenia* Martínez & Carcavallo, 1977, *Belminus* Stål, 1859, *Microtriatoma* Prosen & Martínez, 1952, *Parabelminus* Lent, 1943, *Cavernicola* Barber, 1937, *Psammolestes* Bergroth, 1911, *Rhodnius* Stål, 1859, *Eratyrus* Stål, 1859, *Panstrongylus* Berg, 1879, and *Triatoma* Laporte, 1832 [7]. *Rhodnius*, *Triatoma* and *Panstrongylus* are the most important from an epidemiological point of view [13].

The genera *Rhodnius* and *Triatoma* have been considered paraphyletic [13]. *Panstrongylus* was initially considered monophyletic based on morphological data [2]; however, Marcilla et al. [14], using the internal transcribed spacer 2 (ITS-2) nuclear marker, suggested that *Panstrongylus* was polyphyletic. Later, several phylogenetic analyses indicated this genus is paraphyletic once species of *Panstrongylus* are grouped with species of *Nesotriatoma* Usinger, 1944, and *T. tibiamaculata* (Pinto, 1926) [13, 15–17].

*Triatoma tibiamaculata* is distributed in ten Brazilian states [7] and has epidemiological importance as it has already been found infecting household areas [18] and colonizing peridomiciliar environments [19]. The taxonomy of this triatomine was quite unstable because Pinto [20], based only on morphological characteristics, initially described this species in the genus *Eutriatoma* Pinto, 1926, highlighting that it had intermediate characteristics between *Rhodnius* and *Triatoma*. Later, the species was transferred from the genus *Eutriatoma* to *Triatoma* [21, 22].

Although grouped in *Triatoma*, the phylogenetic position of *T. tibiamaculata* in relation to the other species of this genus has always been uncertain once this triatomine was grouped in all phylogenies with the genus *Panstrongylus* [13, 15–17], rescuing *T. tibiamaculata* and *P. megistus* (Burmeister, 1835) as sister species [13, 16, 17]. Based on this, Gardim et al. [16] suggested a review of the generic status of *T. tibiamaculata*, highlighting that this species possibly belongs to *Panstrongylus*.

Thus, we evaluated the generic status of *T. tibiamaculata* through phylogenetic and chromosomal analysis.

## Methods

### Type of material examined

*Eutriatoma tibiamaculata* Pinto, 1926, syntype. Determined: Pinto, C. 1926, Collected: Travassos, L. 16.XII.1926., Location: Angra dos Reis, Rio de Janeiro, Brazil, deposited in the Entomological Collection of the Instituto Oswaldo Cruz (CEIOC), Rio de Janeiro, Brazil.

### Molecular analysis

For molecular analysis, the genomic DNA of five specimens of *P. lignarius* (Walker, 1873) (from Porto Velho, Rondônia, Brazil), *P. lutzi* (Neiva & Pinto, 1923) (from Irecê, Bahia, Brazil) and *T. tibiamaculata* (from Mogi Guaçu, São Paulo, Brazil) was extracted from gonads using the DNeasy Blood and Tissue kit (QIAGEN®). Amplification of the fragments was performed by polymerase chain reaction (PCR), using primers targeting cytochrome b (*cytb*) and internal transcribed spacer 1 (ITS-1), as described in the literature [23, 24]. The amplified PCR products were visualized by electrophoresis in 1% agarose gel and later purified using the GFX PCR DNA & Gel Band Kit (GE Healthcare and Life Technology®) according to the manufacturer's instructions. Subsequently, this material was submitted for direct sequencing on an ABI 3730 DNA Analyzer (Life Technologies) sequencer from the Research Center on the Human Genome and Stem Cells, University of São Paulo (USP), Brazil.

The gene sequences obtained were grouped with sequences of several molecular markers for 17 taxa available in GenBank (Table 1), which were aligned in the MEGA X program [25] using the Muscle method [26]. For the alignment of ITS-1 and ITS-2, the sequences of the *brasiliensis* subcomplex species are only available concatenated (Table 1); thus, the sequences for the other species had been previously concatenated and then aligned with species of the *Brasiliensis* subcomplex (representatives of the *Triatoma* genus of the *Brasiliensis* subcomplex were used in the phylogeny because *T. tibiamaculata* was initially considered in this subcomplex based on morphological data and geographic distribution [16]).

**Table 1:** GenBank accession number for each marker used in the phylogenetic analysis

Species	Molecular markers								
	*16S*	*18S*	*28S*	*cytb*	*COI*	*COII*	ITS-1	ITS-2	*12S*
***Panstrongylus* genus**									
* P. chinai*				JX400960				AJ306547	
* P. geniculatus*	AF394593		KX109907	KX109903			AM949585	AJ306543	
* P. howardi*				JX400969				JX400871	
* P. lignarius*	AY185833	JQ897584	KX109906	**ON262111**	AF449141			AJ306549	AY185818
* P. lutzi*	KC248969		KC249135	KC249227	KC249307	KC249401	**ON262110**		
* P. megistus*	KC248975	AJ243336	KC249141	KC249232	KC249312	KC249403	AM949580	AJ306542	AF021178
* P. rufotuberculatus*	KY748239	AJ421955		JX400989				AJ306546	
* P. tibiamaculatus* comb. nov	KC249080	KC249127	KC249214	KC249296	KC249389	KC249485	**ON262109**		AY185829
* P. tupynambai*	KC248978		KC249142	KC249234		KC249404			
*Brasiliensis* subcomplex									
* T. brasiliensis*	KC248985	AJ421957	KC249145	KC249239	KC249318	KC249413	KJ125138		AF021187
* T. bahiensis*				KT347298					
* T. juazeirensis*	KC249026		KC249173	AY494169	KF826892		KJ125150		
* T. lenti*	KY576788			KY576789	KY576791				
* T. melanica*	KC249041		KC249183	AY336527	KC249041	KC249461	KJ125147		
* T. petrocchiae*	KY654073			KY654075	KY654074				KY654072
* T. sherlocki*	EU489057		KC249205	EU489058	KC608987	KC249478	KJ125149		
Outgroup									
* Rhodnius prolixus*		AJ421962	AF435860	AF045718	AF449138			AJ286888	AF394519

Bold: Sequences obtained in this study

The alignments were concatenated by name using the Seaview4 program [27], resulting in an alignment with 7993 nucleotides, which was converted in Mesquite 3.2 [28]. Data were partitioned for each molecular marker, and the best model for each one (lowest Akaike information criterion value) was determined in the jModeltest 2 program [29] (Table 2). For the phylogenetic reconstruction by Bayesian inference, the data were submitted to MrBayes 3.2 [30] in an analysis with 100 million generations. Trees were sampled every 1000 generations in two independent runs (each with four Markov chains) and burn-in adjusted to 25%. Tracer v. 1.7 [31] was used to verify the stabilization (ESS values > 200) of the sampled trees, and the generated phylogenetic tree was visualized and edited in the FigTree v.1.4.4 program [32].

**Table 2:** Substitution models for each marker

Molecular markers	Substitution models
*16S*, *cytb*	GTR + I + G
*12S*, *28S*, *COI*, *COII*	GTR + G
*18S*	HKY + I
ITS-1 + ITS-2	HKY + G

### Cytogenetic analysis

*Triatoma tibiamaculata* (from Mogi Guaçu, São Paulo, Brazil), *P. megistus* (from Araraquara, São Paulo, Brazil), *P. lignarius* (from Porto Velho, Rondônia, Brazil) and *P. lutzi* (from Irecê, Bahia, Brazil) males were dissected; the testes were removed and stored in methanol:acetic acid solution (3:1). Slides were prepared by the cell crushing technique (as described by Alevi et al. [33]), and cytogenetic analyses were applied to confirm the karyotype of the species using the lacto-acetic orcein technique [33, 34]. The slides were examined using Jenaval light microscopy (Zeiss) coupled to a digital camera and the Axio Vision LE 4.8 image analyzer system, with a 1000-fold increase.

## Results

### Phylogenetic analysis

**Fig. 1:**
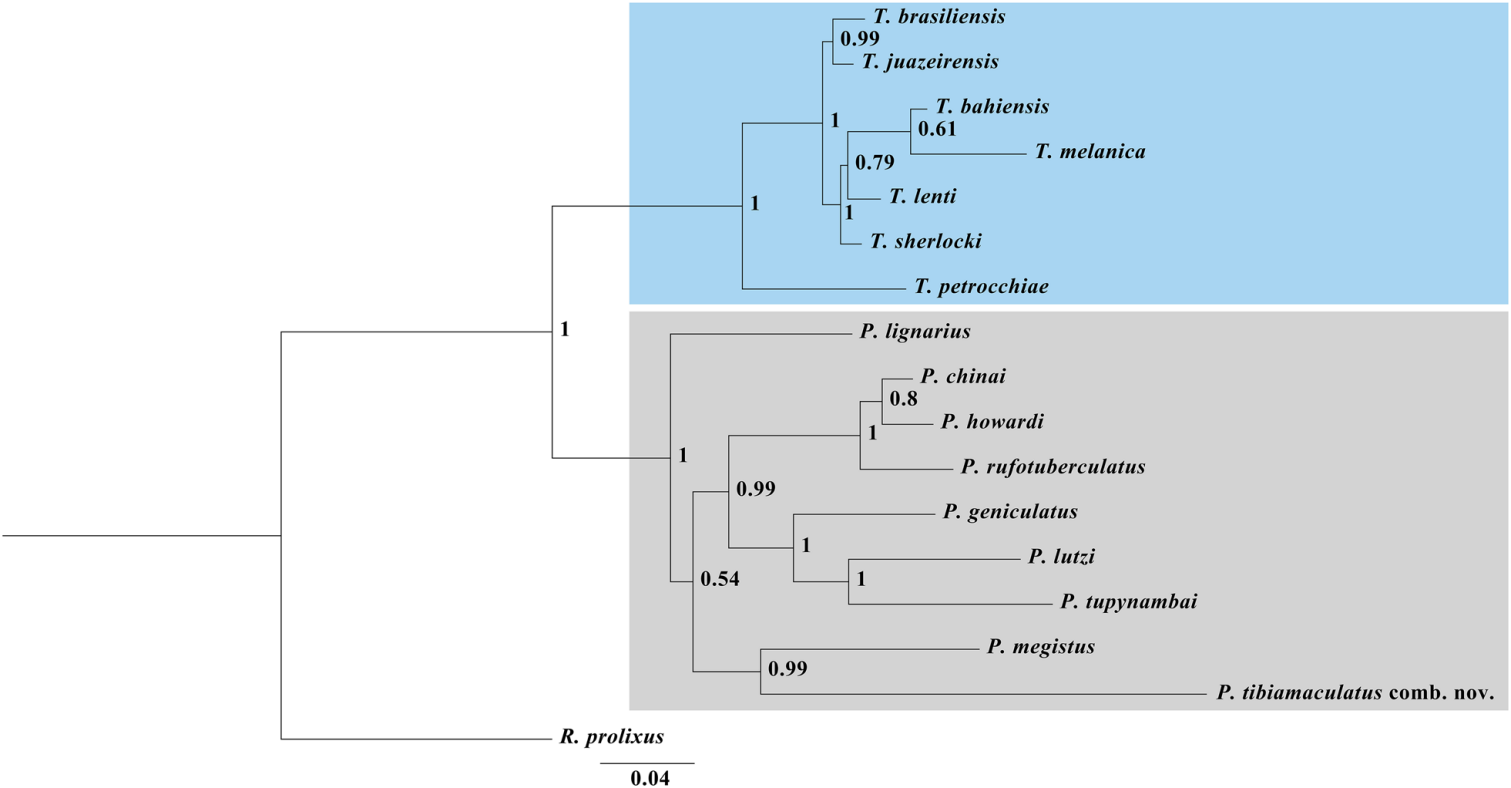
Phylogeny obtained by Bayesian approach. *Rhodnius prolixus* was placed as outgroup. The number in the nodes indicates the posterior probability (> 0.5)

Phylogenetic reconstruction with *cytb* and ITS-1 combined with several mitochondrial and nuclear DNA sequences was deposited in GenBank (*16S*, *18S*, *28S*, *COI*, *COII*, ITS-2 and *12S*) rescued *T. tibiamaculata* with *Panstrongylus* spp. (Fig. 1) in a clade distinct from *Triatoma* spp., demonstrating that *T. tibiamaculata* is a species of *Panstrongylus*.

### Chromosomal analysis

The confirmation of the karyotype of the species *T. tibiamaculata*, *P. megistus*, *P. lignarius* and *P. lutzi*, when combined with literature data [35–40], demonstrates that, except for *P. megistus* and *P. lutzi*, *T. tibiamaculata* and all other species of *Panstrongylus* have the same diploid chromosome set (2n = 23 chromosomes) (Table 3). In addition, based on FISH data in the literature, *T. tibiamaculata* and all species of *Panstrongylus* present markings in a pair of autosomes [41–43] (Table 3), confirming that *T. tibiamaculata* is a species of *Panstrongylus*.

**Table 3:** Cytogenetic characteristics of *P. tibiamaculatus* comb. nov. and *Panstrongylus* spp.

*Panstrongylus* spp.	Karyotype	Autosomal number	Sex determination system	FISH (45S rDNA)
*P. chinai*	2n = 23^a^	20^a^	X_1_X_2_Y^a^	The largest autosomal par^g^
*P. geniculatus*	2n = 23^a^	20^a^	X_1_X_2_Y^a^	The largest autosomal par^h^
*P. howardi*	2n = 23^b^	20^b^	X_1_X_2_Y^b^	The largest autosomal par^i^
*P. lignarius*	2n = 23^a^	20^a^	X_1_X_2_Y^a^	The largest autosomal par^g^
*P. lutzi*	2n = 24^c,d^	20^c^^,d^	X1X_2_X_3_Y^c,d^	The largest autosomal par^i^
*P. megistus*	2n = 21^e^	18^e^	X_1_X_2_Y^e^	The largest autosomal par^g^
*P. rufotuberculatus*	2n = 23^a^	20^a^	X_1_X_2_Y^a^	The largest autosomal par^h^
*P. tibiamaculatus* comb. nov	2n = 23^f^	20^f^	X_1_X_2_Y^f^	The largest autosomal par^g^
*P. tupynambai*	2n = 23^f^	20^f^	X_1_X_2_Y^f^	–

*X* X sex chromosome, *Y* Y sex chromosome

^a^Crossa et al. [35]

^b^Panzera et al. [36]

^c^Santos et al. [37]

^d^Alevi et al. [38]

^e^Schreiber and Pellegrino [39]

^f^Panzera et al. [40]

^g^Panzera et al. [41]

^h^Pita et al. [42]

^i^Panzera et al. [43]

### Generic transfer

**Fig. 2:**
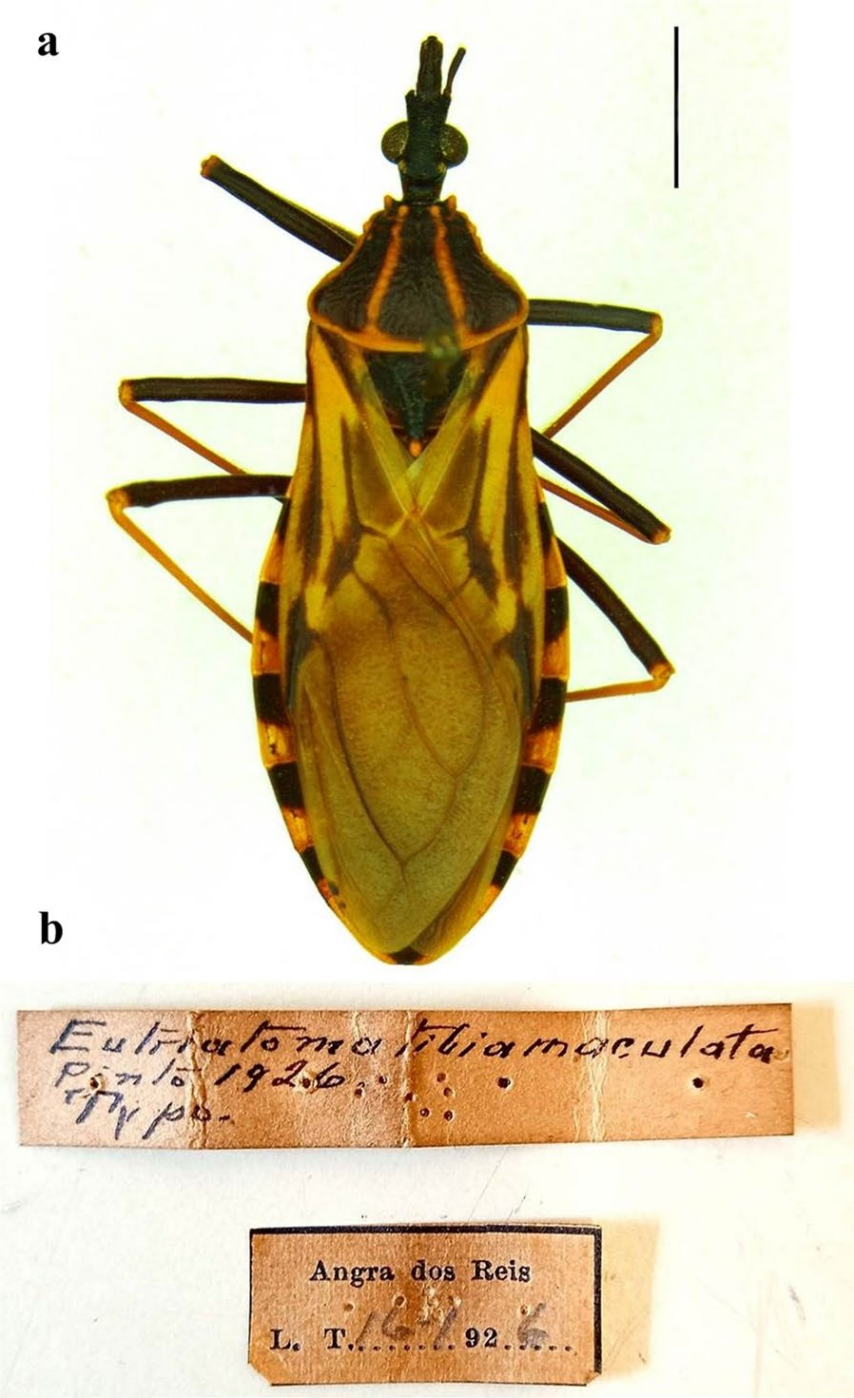
*Eutriatoma tibiamaculata* Pinto, 1926 syntype. **a** Type specimen (dorsal view); **b** labels referring to collection, location and type determination. Bar: 6 mm

Kingdom Animalia Linnaeus, 1758, Phylum Arthropoda von Siebold, 1848, Class Insecta Linnaeus, 1758, Order Hemiptera Linnaeus, 1758, Suborder Heteroptera Latreille, 1810, Family Reduviidae Latreille, 1807, Subfamily Triatominae Jeannel, 1919, Tribe Triatomini Jeannel, 1919, Genus *Panstrongylus* Berg, 1879, Species *Panstrongylus tibiamaculatus* (Pinto, 1926) comb. nov. (Fig. 2).

*Eutriatoma tibiamaculata* Pinto, 1926 (p. 134, Figs. C–E [20]).

*Triatoma (Eutriatoma) tibia-maculata* (Lima, 1940) (p. 199, Fig. 383 [22]).

*Triatoma tibiamaculata* (Pinto, 1926) (p. 902, Fig. 2 [21]).

*Panstrongylus*: the genus name comes from the Greek “*pan*” means whole, and “*strongylus*” means round, plump, burly, a reference to the insect’s robust, rounded body [44].

*tibiamaculatus*: the specific epithet comes from the Latin “*tibia*” and “*maculatus*,” and the combination means stained tibias, a reference to the insect's tibiae being totally "stained" in orange [44].

The change of the specific epithet “*tibiamaculata*” to “*tibiamaculatus*” was carried out based on Art. 31.2 of the International Code of Zoological Nomenclature (ICZN) [45] since “*Panstrongylus*” is masculine—because (i) the ending '-us' usually indicates masculine words; (ii) the ICZN requires that the specific epithet be of the same grammatical gender as the generic epithet, for example, the species of the genus *Panstrongylus* are all male, as *P. geniculatus* (Latreille, 1811), *P. lignarius* and *P. rufotuberculatus* (Champion, 1899), and so is the genus; (iii) the Portuguese versions of Latin words retain the grammatical gender: if the term “*strongyl*” is masculine, so is *Panstrongylus* [46]—and “*tibiamaculatus*” is a latinized adjective.

## Discussion

The chromosomal and phylogenetic relationship of *Panstrongylus tibiamaculatus* comb. nov. and *Panstrongylus* spp. confirms the change of generic status to this species. Thus, the genus *Panstrongylus* includes 16 species now, namely, *P. chinai* (Del Ponte, 1929), *P. diasi* Pinto & Lent, 1946, *P. geniculatus*, *P. guentheri* Berg, 1879, *P. hispaniolae* Poinar, 2013 (fossil species), *P. howardi* (Neiva, 1911), *P. humeralis* (Usinger, 1939), *P. lenti* Galvão & Palma, 1968, *P. lignarius*, *P. lutzi*, *P. martinezorum* Ayala, 2009, *P. megistus*, *P. mitarakaensis* Bérenger & Blanchet, 2007, *P. rufotuberculatus*, *P. tibiamaculatus* comb. nov. and *P. tupynambai* Lent, 1942 [3].

As already mentioned, since 2002, phylogenetic studies have shown the relationship between *P. tibiamaculatus* comb. nov. and *Panstrongylus* spp. (more specifically, *P. megistus*) [13, 15–17] demonstrating that these taxa share common ancestry. Justi et al. [17], based on phylogenetic reconstruction associated with geological events, suggested that the ancestral population that gave rise to *P. tibiamaculatus* comb. nov. and *P. megistus* was distributed along the former connection between the Amazon Forest and the Atlantic Forest and, later, with the climate changes caused by the Andean uplift that resulted in the disappearance of this connection, a vicariance event that resulted in the speciation of *P. tibiamaculatus* comb. nov. and *P. megistus*.

Considering the phylogenetic relationship between *P. tibiamaculatus* comb. nov. and *Panstrongylus* spp. (more specifically, *P. megistus*) [13, 15–17], Monteiro et al. [5] highlight that these species probably descend from a common ancestor that colonized the moist Atlantic forests of eastern Brazil south of parallel 7S. The authors signaled that *P. megistus* is widespread across the Atlantic forests but also occurs in gallery forests throughout the drier Cerrado and stretches into the semiarid Caatinga, the Chaco and parts of the Pantanal and Uruguayan savannahs. On the other hand, Monteiro et al. [5] pointed out that *P. tibiamaculatus* comb. nov. is associated with palms and bromeliads along a narrow strip of coastal Brazil including the Pernambuco, Bahia and Serra do Mar coastal moist forests.

Gardim et al. [16] evaluated ecoepidemiological issues related to *P. tibiamaculatus* comb. nov. and *P. megistus*. The authors also emphasized that the close relationship between *P. megistus* and *P. tibiamaculatus* comb. nov. may help to explain the recent finding of the latter species invading human domiciles in downtown Salvador, Bahia State, Brazil.

Justi et al. [17] grouped the species of *Panstrongylus* into two groups: *geniculatus* and *megistus*. However, more recently Monteiro et al. [5] considered four groups: *P. rufotuberculatus*, *P. lignarius*, *P. geniculatus* and *P. megistus*. Our results also retrieved four groups, namely, *P. rufotuberculatus* (composed of *P. chinai, P. rufotuberculatus* and *P. howardi*), *P. lignarius* (composed of *P. lignarius*), *P. geniculatus* (composed of *P. geniculatus, P. lutzi* and *P. tupynambai*) and *P. megistus* (composed of *P. megistus* and *P. tibiamaculatus* comb. nov.).

Although *P. tibiamaculatus* comb. nov. has morphological characteristics that approximate it to *Triatoma* spp. (which led to the misclassification of the species in this genus), the most prominent morphological feature that distinguishes the genus *Panstrongylus* from other triatomines is the short head, with antennae close to the eyes [3]. The geometric morphometric of head, for example, is a tool that discriminated *Panstrongylus* and *Triatoma* based on the position of the antennal insertion relative to the eyes [47]. Justi et al. [12] highlighted that the morphological divergences observed between *P. tibiamaculatus* comb. nov. and the other *Panstrongylus* may be due to morphological convergence with *Triatoma* spp., because variations in the size of the eyes of *Panstrongylus* spp. have already been reported in the literature [48], and these variations influence the distances between the antennas and the eyes.

Some morphological similarities between *P. tibiamaculatus* comb. nov. and the species in the *brasiliensis* subcomplex led Schofield and Galvão [49] to group these species in this complex. However, based on chromosomal divergences, Alevi et al. [33] proposed the exclusion of the species from this complex. From a karyosystematic point of view, while *P. tibiamaculatus* comb. nov. has 2n = 23 chromosomes (which approximates it to most species of *Panstrongylus*), all South American *Triatoma* species have 2n = 22 (species of the *Brasiliensis, Infestans, Maculata, Pseudomaculata, Rubrovaria* and *Sordida* subcomplexes) or 24 chromosomes (*Vitticeps* subcomplex species) [50]. Based on the ancestral karyotype of Triatominae (2n = 22) [51], Alevi et al. [52] suggested that during the divergence of the common ancestor of *Panstrongylus* there was a fission in sex chromosome X, which resulted in the karyotype 2n = 23 (karyotype shared by *P. chinai, P. geniculatus, P. howardi, P. lignarius, P. rufotuberculatus, P. tibiamaculatus* comb. nov. and *P. tupynambai*). However, the authors suggested that during the karyotypic evolution of *Panstrongylus*, two possible punctual events occurred: fusion in a pair of autosomes in *P. megistus* and fission in the sex chromosome X in *P. lutzi*. The karyotypes of *P. megistus* and *P. lutzi* (2n = 21 and 2n = 24, respectively) were observed only in five species of *Triatoma* (*T. nitida* Usinger, 1939, *T. eratyrusiformis* Del Ponte, 1929*, T. melanocephala* Neiva & Pinto, 1923*, T. vitticeps* (Stål, 1859) and *T. breyeri* Del Ponte, 1929 [52]), suggesting that these evolutionary events occurred independently during the chromosomal evolution of triatomines.

In addition, *P. tibiamaculatus* comb. nov. and all other *Panstrongylus* species (regardless of the number of chromosomes) have 45S rDNA probes restricted to a pair of autosomes [41–43]. Pita et al. [53] suggest that the chromosomal position of 45S rDNA is variable in Triatominae, although it is conserved among closely related species (such as *P. tibiamaculatus* comb. nov. and *Panstrongylus* spp.). In addition to the genetic relationships observed between *P. tibiamaculatus* comb. nov. and *Panstrongylus* spp., morphological similarities between fifth-instar female nymphs of *P. megistus* and *P. tibiamaculatus* comb. nov. (more specifically in the structures of the eighth ventral segment as well as between setae) were observed [54]. Furthermore, Nascimento et al. [55] also observed similarities between spermathecae morphology from *P. lignarius, P. megistus* and *P. tibiamaculatus* comb. nov., and Mello et al. [56] recorded a relationship between exocorial cells in eggs of *P. tibiamaculatus* comb. nov. with *Panstrongylus*.

## Conclusion

Thus, based on chromosomal and phylogenetic characteristics, we state that *P. tibiamaculatus* comb. nov. belongs to the genus *Panstrongylus* and that the morphological features shared with *Triatoma* spp. represent homoplasies.

## Data Availability

GenBank accession numbers of sequences generated in this study: *P. tibiamaculatus* ITS-1 (ON262109), *P. lutzi* ITS-1 (ON262110) and *P. lignarius cytb* (ON262111).
